# Increased PDT Efficacy When Associated with Nitroglycerin: A Study on Retinoblastoma Xenografted on Mice

**DOI:** 10.3390/ph15080985

**Published:** 2022-08-10

**Authors:** Carole D. Thomas, Mihaela Lupu, Florent Poyer, Philippe Maillard, Joël Mispelter

**Affiliations:** 1 Institut Curie, Bât. 112, Centre Universitaire, 91405 Orsay, France; 2 U1288 INSERM, LITO Laboratoire d’Imagerie Translationnelle en Oncologie, Bât. 101B, Centre Universitaire, 91405 Orsay, France; 3 Université Paris-Sud, 91405 Orsay, France; 4 U1196 INSERM, Bât. 112, Centre Universitaire, 91405 Orsay, France; 5 UMR 9187 CNRS, Bât. 112, Centre Universitaire, 91405 Orsay, France

**Keywords:** nitroglycerin ointment, PDT, retinoblastoma, sodium MRI, mice

## Abstract

Purposes: The aim of the study was to assess the efficacy of a treatment protocol that combines photodynamic therapy (PDT) and nitroglycerin (NG) on human retinoblastoma tumors xenografted on mice. We aimed to increase the PDT efficiency (in our least treatment-responsive retinoblastoma line) with better PS delivery to the tumor generated by NG, which is known to dilate vessels and enhance the permeability and retention of macromolecules in solid tumors. Methods: In vivo follow-up of the therapeutic effects was performed by sodium MRI, which directly monitors variations in sodium concentrations non-invasively and can be used to track the tumor response to therapy. NG ointment was applied one hour before PDT. The PDT protocol involves double-tumor targeting, i.e., cellular and vascular. The first PS dose was injected followed by a second one, separated by a 3 h interval. The timelapse allowed the PS molecules to penetrate tumor cells. Ten minutes after the second dose, the PS was red-light-activated. Results: In this study, we observed that the PDT effect was enhanced by applying nitroglycerin ointment to the tumor-bearing animal’s skin. PDT initiates the bystander effect on retinoblastomas, and NG increases this effect by increasing the intratumoral concentration of PS, which induces a higher production of ROS in the illuminated region and thus increases the propagation of the cell death signal deeper into the tumor (bystander effect).

## 1. Introduction

Photodynamic therapy (PDT) is based on the activation of a non-mutagenic photosensitizing agent (PS) by illuminating the targeted tissues with visible light. PDT is a relatively recent approach for the treatment of small tumors on the surface or accessible by endoscopy in which the photosensitizing agent absorbs light and generates cytotoxic reactive oxygen species (ROS), leading to cell death through apoptosis and/or necrosis [1,2]. PDT is not only used for cancers; it is also used for various pathologies, such as AMD (Age-Related Macular Degeneration) [3], acne [4], Barrett’s esophagus [5], warts [6] and actinic keratosis [7].

PDT is a highly selective therapy. It relies on the simultaneous presence of three components: the photosensitizing agent (PS), molecular oxygen and visible or near-infrared (NIR) light, none of which is toxic or damaging to cells/tissues on its own. The destruction of unwanted cells and tissues such as tumors by PDT is achieved by using visible or near-infrared radiation to activate a PS, a light-absorbing agent that, in the presence of molecular oxygen, results in the production of singlet oxygen and other reactive oxygen species. These cytotoxic species damage and kill target cells and are responsible for cytotoxicity in neoplastic cells and tumor regression. The photochemical effect itself is very rapid and localized only where light meets the PS [8,9]. Light absorption triggers very rapid photochemical reactions that generate cytotoxic products such as singlet oxygen or other ROS when the appropriate wavelength encounters the PS.

ROS induce biological damage in the targeted cells and are responsible for the most important therapeutic effects. Two main pathways are involved in the therapeutic effect of PDT: the first one is direct and local, where ROS have been produced; the second is indirect by activating and transmitting cell death signaling signals long after the first step. Therefore, two cell death pathways may be involved here: a rapid one characterizing the first step, where PS activation directly induces ROS formation, and a second, longer-scale one, where cell death cascade signals are involved in the killing process (the bystander effect). The bystander effect in PDT is the propagation of cellular death beyond the initial light penetration depth and beyond the area of light penetration into the tumor and may result from cell-to-cell signaling. In previous studies, we reported evidence that PDT induced a bystander effect in human colorectal and xenografted retinoblastoma tumors [10,11]. The extensive cell killing is a combined process of direct cellular photodamage followed by the long-time-scale propagation of cell death: the bystander effect. Among the different factors involved in PDT efficiency, we demonstrated that different outcomes depended on the initial cell density of the tumor (the lower the cell density, the less effective the treatment) for three human retinoblastoma cell lines xenografted in mice [12]. 

Many studies have indicated that PDT may not be sufficient to completely eradicate the tumor, suggesting that despite very promising results, this treatment does not destroy the totality of the tumor, and there is often tumor regrowth after the end of the treatment [13,14,15]. As mentioned above, intratumoral oxygen and the amount of PS are important factors for PDT. Different strategies are used to improve tumor oxygenation [16], for instance, the use of oxygen carriers with perfluorocarbon nanoparticles [17], liposome-encapsulated hemoglobin [18], hyperbaric oxygen [19] and oxygen self-supplied enzyme nanogels [20]. In PDT, the amount of PS in the tumor cells is an important factor. The PS first moves into the vascular system, extravasates and eventually enters the tumor cells. We propose increasing the efficiency of PDT by enhancing the release of PS into the tumor through the action of nitroglycerin (NG).

NG, a nitric oxide (NO) donor, has been used in clinical practice for over a century in cardiology to prevent angina (chest pain) caused by coronary artery disease, and it is well tolerated [21]. However, more recently, NG was also found to improve drug delivery [22,23,24]. NO is a gaseous molecule that plays a unique role as a chemical messenger involved in vasodilator, neurotransmitter and anti-platelet activities. NG is administered topically or orally, and the active molecular species generated is NO, which is converted to nitrite and then to NO. 

Seki, Fang and Maeda demonstrated that with NG, the extravasation of macromolecules into tumor tissue is significantly improved, and they named this phenomenon the enhanced permeability and retention (EPR) effect of macromolecules in solid tumors [22,25,26]. NG is effective regardless of its site of application, and thus, NG improves drug delivery to tumors and therapeutic effects [22,26]. In addition, they showed that NG significantly increased blood flow to tumors [22]. Similarly, Yasuda et al. reported that NG increased the sensitivity of tumors to chemotherapy by increasing blood flow in hypoxic tumors [27]. Rapozzi et al. studied the effect of nitric oxide on cancer treated with PDT in vitro and in vivo [28]. In particular, they tested the combined effect of a nitric oxide donor (DETA/NO) and PDT on C57BL/6 mice bearing a syngeneic B78-H1 melanoma. They observed that the combined treatment delayed tumor growth more effectively than each individual treatment. They noted that the efficacy of PDT was enhanced when the PS was used in combination with an NO donor. To our knowledge, there are no studies regarding the enhancement of the EPR effect or PDT by an NO donor (NG) in retinoblastomas (Rb).

In this context, we aimed to combine the PDT protocol with nitroglycerin on Rb xenografted tumors. Rb is a cancer that develops from immature cells of the retina and is the most common malignant tumor of the eye in children. The disease is fatal if left untreated, whereas the survival rate is over 95% in developed countries if treated appropriately. All clinically used treatments are characterized by short- and long-term side effects [29,30,31]. Non-mutagenic therapies such as PDT are of considerable clinical interest, taking into consideration the early age of the patients. In the present work, one of three Rb lines—the one that was the least responsive to treatment—was chosen to be treated with the combination of PDT plus NG [12]. 

In vivo follow-up of the therapeutic effects was performed using sodium MRI, which allows direct monitoring of changes in sodium concentrations non-invasively and can be used to assess tumor response to treatment [12,32].

The aim of our study was to determine whether PDT combined with nitroglycerin ointment is able to induce major cell death in a human Rb line that has shown the poorest results in terms of tumor destruction by PDT alone [12].

## 2. Results

**Figure 1:**
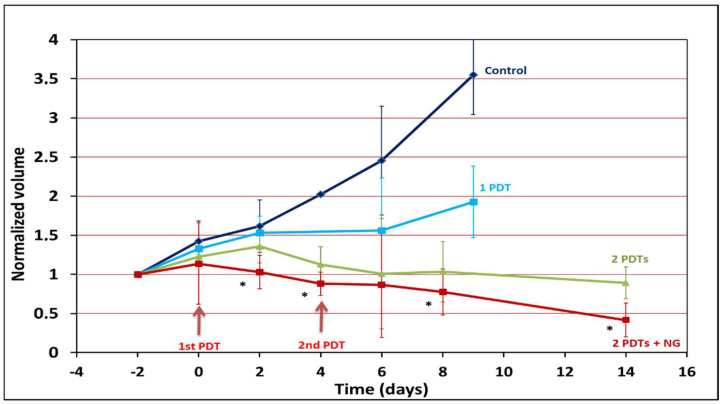
Variation in the relative tumor volume over the time course of the experiment, determined by manual delimitation on proton MRI images. Dark blue line: control; blue line: 1PDT; green line: 2PDTs; red line: 2PDTs + NG. * *p* < 0.05 (comparison between the 2PDT group and 2PDTs + NG group). Compared to the control group, all treatments are efficient in reducing tumor growth. The combination of NG and PDT dramatically decreases the tumor volume below its initial value.

Four animal groups were followed in this study. Two animal groups (control and 1PDT) were followed for 9 days, and two groups (2PDTs and 2PDTs + NG) were followed for 14 days (Figure 1). All treatments were efficient in reducing tumor growth a few days after PDT compared to the control. However, after a single treatment, regrowth of the tumor was observed (almost twice the initial volume on the 9th day). In contrast, after two PDTs, the arrest of tumor growth was observed and remained constant until the end of the follow-up. When NG was applied topically 1 h before the two PS injections, a significant decrease in tumor volume was observed compared to the same treatment without NG. Furthermore, the combination of NG and PDT decreased the tumor volume below its initial value. For two animals, the tumors were completely damaged at the end of the follow-up. Follow-up was performed by ^1^H/^23^Na imaging in order to determine the tumor volume evolution (^1^H MRI) as well as the treatment outcome (^23^Na MRI).

**Figure 2:**
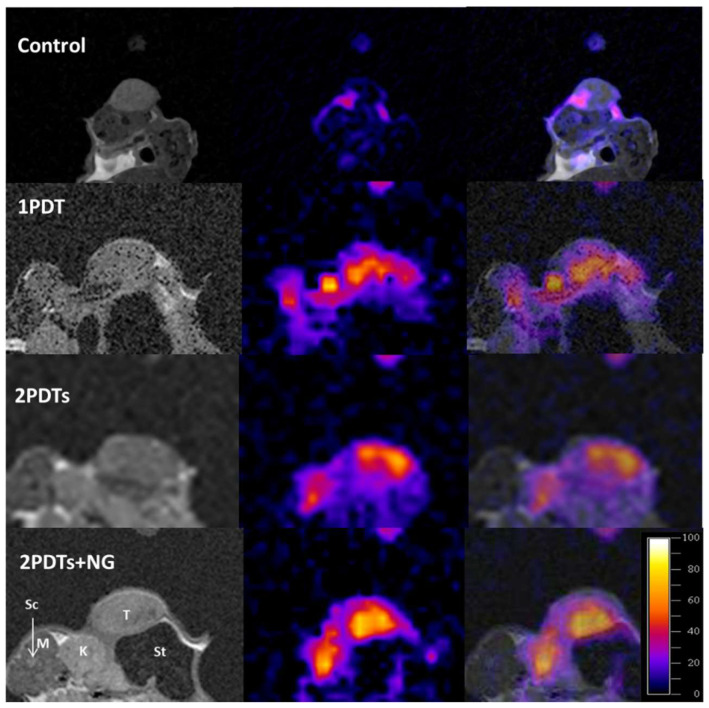
Proton and sodium MRI images obtained on mice with xenografted retinoblastoma tumors treated with PDT and nitroglycerin. Proton images with anatomical details (**left**), the corresponding sodium images (**middle**) and the superposition of the two images (**right**). First line, control; second line, 1PDT; third line, 2PDTs; and fourth line, 2PDT + NG. Sc: spinal cord; K: kidney; St: stomach; M: muscle; T: tumor. In sodium images, tissues with very high sodium content (kidney and tumor) appear with high contrast. In the tumor, it reflects damage, such as necrosis, induced in the light penetration pathway into the tissue.

The different MRI images obtained in the four groups of animals are presented in Figure 2: the proton image (left column), the corresponding sodium image (middle column) and a superposition of both images (right column). The superposition of the images allows better anatomical localization of the sodium content. Anatomical details are shown for the 2PDTs + NG group in the proton image. In the sodium image, only the tissues with a very high sodium content appear with high contrast: the tumor and the kidney (which is the site of urinary sodium excretion).

Sodium imaging, as performed in our study, revealed the treatment-induced changes in tumors. After cell death, the volume occupied by the damaged cells was replaced by the extracellular compartment even before changes in the tumor dimensions were observed.

Just after the first PDT (about 2 h), we observed an increase in the high-sodium signal in a region compatible with red light penetration into the tissue. More interestingly, after the second PDT treatment, this area expanded to the whole tumor, beyond the depth of light penetration.

When NG was applied topically, a marked increase in sodium signal intensity was observed after PDT (Figure 2). This increase was higher than that observed in the PDT-only group. Images from the control group are not shown, as the tumor sodium intensity was constant throughout the follow-up.

**Figure 3:**
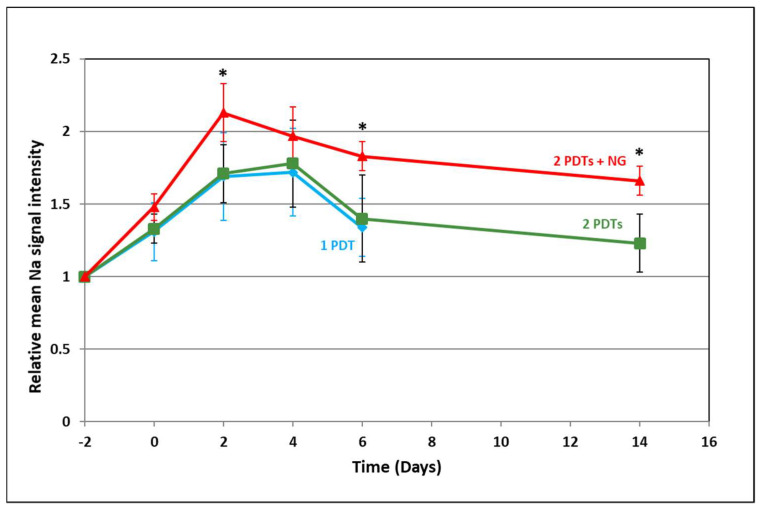
Evolution of the relative mean sodium signal intensity. Blue line: 1PDT; green line: 2PDTs; and red line: 2PDTs + NG. The sodium content after one or two PDTs is similar, whereas for the group that received two PDTs with NG, the sodium content is higher, indicating large extracellular space and considerable cellular damage. * *p* < 0.05.

Changes in signal intensity were analyzed on a central slice of the tumor. The sodium signal intensity was always higher for the group with NG than without (one or two PDTs), and this difference was maintained throughout the observed period (Figure 3). This sodium signal intensity did not change during the follow-up in the control group.

**Figure 4:**
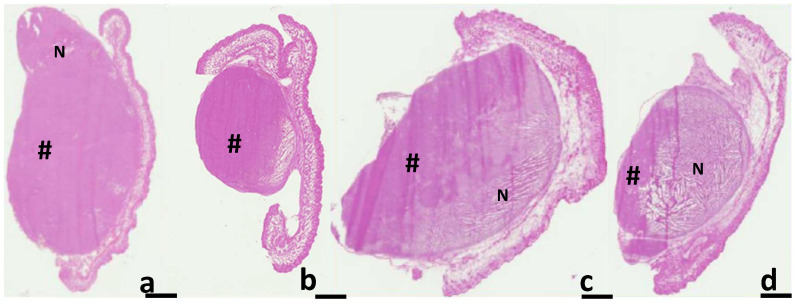
Histological slides at the end of the follow-up. (**a**): Control, (**b**): 1PDT, (**c**): 2PDTs and (**d**): 2PDTs +NG. For the control group, there was very little spontaneous necrosis at the end of the follow-up, whereas damage was more substantial for the 1 and 2 PDT groups and higher for the 2PDT group treated with NG. N: necrotic area; #: non-necrotic area. Scale bars: 1 mm.

At the end of the follow-up, histological examination was performed to compare the effects of the different treatments on the tumor structure (Figure 4). On the histological slides stained by H&E, important differences between the four groups were detected. In addition, macroscopic cell destruction was visible in the 2PDTs + NG group compared to the other groups.

**Figure 5:**
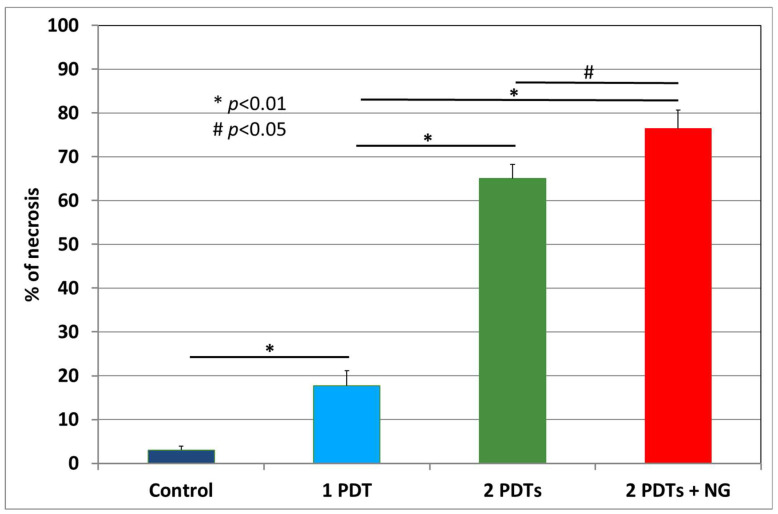
Percentage of necrosis determined by histological examination at the end of the follow-up for the different groups. The group receiving the combination NG plus PDT had significantly more tumor necrosis than the other groups.

The percentage of necrosis at the end of the follow-up indicated that the NG + PDT combination was more effective in terms of tumor damage (77% vs. 65% for 2PDTs alone and 18% for 1PDT) (Figure 5). It should be noted that this retinoblastoma line showed very little spontaneous necrosis (only 3% necrosis in the control group).

## 3. Discussion

Although PDT is very effective under clinical conditions for many dermatological diseases, such as psoriasis, basal cell carcinoma and acne [5,33,34], the results obtained are poorer for some tumors, as shown by several studies on various types of cancers, such as bladder carcinoma, lung cancer and prostate cancer [35,36,37,38]. Therefore, we used tumors that are particularly difficult to treat with PDT; we chose to use the Rb line in which the PDT efficacy was lower compared to the other Rb lines used in our previous study [12]. We assumed in [12] that this poor response to PDT was mainly due to the fact that the cell architecture of this line is sparser than the one that gives the best response. After PDT, cell-to-cell signaling may be blocked, or the first cell death signals induced by PDT are not sufficient to trigger the bystander process.

PDT has many advantages, such as its applicability to multiple diseases, low cost, ease of treatment application, highly localized targeting by light, etc. However, it has potential drawbacks and obstacles to its use [39]. Firstly, the tumor or tissue to be treated must be accessible to an optical fiber and not be too thick for the light to reach the deepest part. Secondly, PDT often generates pain and/or inflammation [37,40], and thirdly, its use requires patients to protect themselves from light rays during the retention time of the PS in the body.

For several years, many teams have been working to improve the results of PDT treatments. Several strategies (non-exhaustive list) have been developed: for example, improving tumor oxygenation [16,17,18,19,20], limiting hypoxia [16,41], combining PDT with other procedures, such as radiotherapy, chemotherapy or surgery [38], combining PDT with other treatments, such as immunotherapy [42], improving the light delivery system with a specific light device, such as a fabric device [43], improving the delivery of PS into the tumor [36], etc. We chose the latter strategy using NG in order to improve the delivery of PS to the tumor.

In our study, we targeted both blood vessels and tumor cells; the delay between the two PS injections (3 h) allows the PS to penetrate the cells (in vitro, it has been shown that this PS penetrates tumor cells [10]), and the illumination of the tumor 10 min after the second injection allows the tumor vasculature to be targeted by the second dose of PS, which has not had time to penetrate the cells and remains in the vasculature.

It seems that the concentration of NO is an important element for either a pro-tumor (low concentration) or anti-tumor (high concentration) effect [44]. Furthermore, it has recently been shown that the action of NO, depending on its concentration, can induce pro- or anti-malignant properties depending on its steady-state levels [45]. The clinical outcome depends on the type and stage of the tumor as well as the tumor microenvironment [46]. In our experiments, retinoblastomas were heterografted subcutaneously in both flanks of nude mice. Only one side was used for treatments, while the other side was maintained as a non-illuminated reference. In the non-illuminated tumor of the NG-treated group, we did not observe a decrease in tumor volume (data not shown). It is likely that under our experimental conditions, the concentration of NO released by NG was too low to observe an effect on tumor growth. In our study, we applied a low dose of ointment (0.2 mg) containing nitroglycerin (4 mg/g) on the skin of the animal one hour before the PDT treatment. Nitroglycerin is very liposoluble and is readily transported through membranes. NG has a plasma half-life of approximately 1 to 4 min; following hepatic and intravascular metabolism, its biologically active metabolites have a half-life of approximately 30–40 min [47].

Some studies showed that a red laser is able to release NO storage from tissue [48,49]. Lohr et al. showed in both purified systems and the myocardium that R/NIR light can decay nitrosyl hemes and release NO, and that this released NO may enhance the cardioprotective effects of nitrite. In smooth muscle, NG is converted to NO, which then activates guanylyl cyclase, which converts guanosine triphosphate (GTP) to guanosine 3′,5′-monophosphate (GMPc) in vascular smooth muscle and other tissues. GMPc then activates numerous protein kinase-dependent phosphorylations, which ultimately results in the dephosphorylation of myosin light chains in smooth muscle fibers. This causes smooth muscle relaxation in the blood vessels, resulting in the desired vasodilator effect. The biological effects of NO depend on many factors, such as the formation of the molecule, its metabolism, the types of nitric oxide synthases present and the concentration of NO present [50].

In this study, we tested the PDT + NG combination therapy by looking for an improvement in the therapeutic outcome of PDT for the least treatment-responsive tumor line. NG was chosen for its EPR properties [22,23,51,52,53]. NG was applied on the ventral skin of the mice based on the work of Seki, who did not observe any influence at the site of application in terms of drug concentration in the tumor [22]. Topical application of NG is thought to improve PS delivery to the tissue by dilating the vascular system. Finally, dilation of the tumor vasculature also increases the amount of PS present in the tumor tissue, which quantitatively increases the ROS generated after activation.

With the combination of NG + PDT, a significant decrease in tumor volume was observed, together with an increase in the percentage of necrosis. It is interesting to note that for two animals, the tumors disappeared completely at the end of the follow-up. In addition, with sodium MRI, we saw a significant increase in sodium content throughout the tumor, indicating an increase in extracellular space related to cell death (apoptosis and/or necrosis). Nitroglycerin increases this effect and, therefore, increases the overall efficacy of the treatment. To explain our results, we hypothesized that NG allows an increase in the PS concentration in tumor cells, with the amount of PS in the tumor being a critical point for PDT treatment. For Seki and Xiang, NG acted by enhancing the EPR effect [22,23]. We propose two ways in which NG can increase PDT efficacy. On one hand, the application of NG locally increases the concentration of PS, thereby producing more ROS in the illuminated area. This enhances the number of cellular death signals that trigger the bystander effect, as already observed in a denser cellular line [12]. On the other hand, the contribution of EPR leads to the same therapeutic effect.

## 4. Materials and Methods

### 4.1. Nitroglycerin, PS and Treatment Protocols

One hour prior to PDT treatment, mice were given a dose of 0.2 mg nitroglycerin ointment (NG) (Rectogesic^®^ 4 mg/g ProStrakan, Neuilly sur Seine, France). This dose was applied topically to the ventral skin of the mice for 5 min.

A glycoconjugated porphyrin derivative was used as a photosensitizing agent. Its absorbance of visible light was tested in order to determine the most suitable wavelength to excite the molecule [54]. In the Q band region, the absorbance peak at 650 nm (red light) was used. The damage induced by PDT is strictly related to PS localization during illumination, hence the double-targeting PDT protocol, as detailed in a previous paper [10].

An i.v. dose (0.6 mg/kg, 0.42 µMol/kg) of PS was followed by a second identical dose, separated by an interval of 3 h (double-targeting PDT). We assumed that this timelapse allows the first dose of PS to penetrate tumor cells or plasma membranes, as observed previously, while the second dose is mainly located in the vascular system. Shortly after the administration of the second dose (10 min), the tumor was exposed to light. Therefore, both the cancer cells and the blood vessels were targeted. For mice receiving two PDTs, there was a four-day interval between treatments.

### 4.2. Laser Illumination

Illumination was performed at 650 nm (red light) using a diode laser with an output power of 100 mW. A number of studies have indicated that, in general, tissue necrosis requires the production of 0.41–0.56 mM singlet oxygen [55]. In terms of oxygen consumption, this represents 6–7 times the metabolic oxygen consumption, thus exceeding the capacity of the microvasculature to supply oxygen to the irradiated tissue [15,56]. The illumination time was fractioned (2 min of light followed by the same period in darkness) to ensure re-oxygenation of the tissue, which is necessary for ROS formation. The total illumination time was determined on the basis of the tumor dimensions following the formula:

$$\mathrm{Time}\;(s)=\frac{\;[\mathrm{Fluence}\;\left(J/{\mathrm{cm}}^{2}\right)\times \;\mathrm{Surface}\;\left({\mathrm{cm}}^{2}\right)\;}{\mathrm{Incident}\;\mathrm{power}\;\left(\mathrm{Watt}\right)}$$

where Fluence = 75 J/cm^2^ and Incident power = 0.1 W.

### 4.3. MRI

Proton and sodium MRI were performed at 4.7 T using a Bruker Biospec small animal MRI scanner. Multi-slice, multi-echo ^1^H images were recorded for localization purposes and tumoral volume determination (respiratory trigger, FOV = 6.8 cm, TE = 12 ms, NE = 10, matrix 256 × 256, and slice thickness 1 mm). Tumor volume was determined using proton MRI images by manual delimitation.

Single-slice, multi-echo (40 echoes) ^23^Na images were recorded for sodium studies (TE = 6.7 ms, FOV = 6.8 cm, matrix 64 × 64, slice thickness 3 mm, and resolution 1 × 1 × 3 mm^3^) using 160 averages. Since anti-tumoral treatment will damage tumor blood vessels and/or cells, resulting in cell death, ^23^Na is used as an endogenous probe to map the extracellular compartment (the most sodium-rich) and to characterize cell density. In tissues with low cell density, the extracellular compartment is larger, and therefore, the average sodium concentration is higher due to the natural gradient of sodium concentration between the intra- and extracellular compartments (up to 15 mM (intracellular)/up to 150 mM (extracellular)). Conversely, in areas with high cell density, the extracellular space is limited, and the local sodium concentration is low. Damage to the vascular system and necrosis or apoptosis decrease cell density and increase the local sodium concentration [12].

### 4.4. Tumor Model and Animal Handling

Adult female Swiss nude/nude mice were purchased from Charles River (Les Oncins, France) and bred in our institute’s animal facility. The experimental procedures were specifically approved by the ethics committee of the Institut Curie: CEEA-IC #118 (authorization number #91-429). The experiments were performed in accordance with the guidelines for animal experimentation (authorization number C91 471 108). Human retinoblastomas (Rb200) provided by the Transfer Department of our institute were initially implanted subcutaneously into nude mice. Solid tumors grown on these donor mice were removed immediately after killing the mouse and then cut into small pieces (about 3 mm^3^). These pieces were heterografted subcutaneously on both flanks of anesthetized mice (Isoflurane 2%). Only one side was used for treatments, and the other side was maintained as an unilluminated reference. PDT experiments were performed 4 weeks after tumor implantation when the tumor size was about 10 mm in diameter. Cohorts of 5 mice were used for all treatment protocols. We used four groups of mice: control, 1PDT, 2PDTs and 2PDTs + NG.

### 4.5. Histology

At the end of the trial, anesthetized mice were killed. Tumors were then removed, embedded in OCT™ (Tissue-Tek^®^, Sakura, Nagano, Japan) and frozen, and several 10 μm thick sections were cut at −20 °C using a microtome (Leica CM3050S, Leica Microsystèmes SAS, Nanterre, France) and stained with hematoxylin and eosin. The sections were scanned at 4000 dpi (Nikon Super Coolscan 8000, Tokyo, Japan). For tumor necrosis estimations, five sections in the center of the tumor were selected and scanned at 4000 dpi. The percentage of necrosis was determined by manual delimitation using Amira software (Mercury Computer Systems; TGS, Bordeaux, France).

### 4.6. Statistical Analysis

Data on relative tumor volume and the percentage of necrosis are presented as mean ± SEM for each group. A Mann–Whitney test was used to compare the different groups, and *p* < 0.05 was considered statistically significant.

## 5. Conclusions

We show that PDT is effective in treating retinoblastomas and that the combination of NG ointment with PDT increases the treatment efficiency for tumor lines that are less responsive to PDT treatment. Indeed, PDT efficacy was enhanced by applying nitroglycerin ointment to the skin of tumor-bearing animals. PDT triggers the bystander effect in retinoblastomas, and NG enhances this effect by increasing the intratumoral concentration of PS, which induces a higher production of ROS in the illuminated region and thus increases the propagation of the cell death signal deeper into the tumor.

The nitroglycerin–PDT combination seems very promising for applying to the clinic in the future, especially for tumors that do not respond well to PDT alone. In addition, this ointment is very easy to use, well tolerated and very cheap. Further developments toward clinical applications are needed.

In conclusion, the use of NG is an easy and inexpensive way to improve the efficacy of PDT, especially for tumors that respond poorly to PDT alone. This gives the PDT + NG combination many important advantages for potential clinical use.

## Data Availability

All data are available on request from the Institut Curie Research Centre.
